# Sexual and gender minority content in undergraduate medical education in the United States and Canada: current state and changes since 2011

**DOI:** 10.1186/s12909-024-05469-0

**Published:** 2024-05-01

**Authors:** Carl G. Streed, Amy Michals, Emily Quinn, John A. Davis, Kylie Blume, Katharine B. Dalke, David Fetterman, Gabriel Garcia, Elizabeth Goldsmith, Richard E. Greene, Jessica Halem, Helene F. Hedian, Isabel Moring, May Navarra, Jennifer Potter, Jennifer Siegel, William White, Mitchell R. Lunn, Juno Obedin-Maliver

**Affiliations:** 1 Section of General Internal Medicine, Department of Medicine, Boston University Chobanian and Avedisian School of Medicine, 801 Massachusetts Ave., Room 2082, Boston, MA 02118 USA; 2 GenderCare Center, Boston Medical Center, Boston, MA USA; 3 Biostatistics & Epidemiology Data Analytics Center, Boston University School of Public Health, Boston, MA USA; 4 Division of Infectious Diseases, Department of Medicine, University of California San Francisco, San Francisco, CA USA; 5 Department of Neurology, University of Wisconsin-Madison School of Medicine and Public Health, Madison, WI USA; 6 Department of Psychiatry, University of Pennsylvania Perelman School of Medicine, Pennsylvania, PA USA; 7 Fetterman & Associates, Hadley, MA USA; 8 Claremont Graduate University, Claremont, CA USA; 9 Division of Gastroenterology and Hepatology, Department of Medicine, Stanford University School of Medicine, Stanford, CA USA; 10 Minneapolis Veterans Affairs Health Care System, Minneapolis, MN USA; 11 Department of Medicine, University of Minnesota Medical School, Minneapolis, MN USA; 12 Division of General Internal Medicine, Department of Medicine, NYU Grossman School of Medicine, New York, NY USA; 13 Eidos LGBTQ+ Health Initiative, University of Pennsylvania, Philadelphia, PA USA; 14 Division of General Internal Medicine, Johns Hopkins University School of Medicine, Baltimore, MD USA; 15 Center for Transgender and Gender Expansive Health, Johns Hopkins, Baltimore, MD USA; 16 Treehouse Pediatrics, Round Rock, TX USA; 17 The Fenway Institute, Fenway Health, Boston, MA USA; 18 Division of General Medicine, Department of Medicine, Beth Israel Deaconess Medical Center, Boston, MA USA; 19 Division of General Internal Medicine, Department of Medicine, Massachusetts General Hospital, Boston, MA USA; 20 Transgender Health Program, Massachusetts General Hospital, Boston, MA USA; 21 San Francisco Emergency Medical Associates, California Pacific Medical Center, San Francisco, CA USA; 22 Department of Emergency Medicine, Harbor-UCLA Medical Center, Los Angeles, CA USA; 23 Division of Nephrology, Department of Medicine, Stanford University School of Medicine, Stanford, CA USA; 24 Department of Epidemiology and Population Health, Stanford University School of Medicine, Stanford, CA USA; 25 Department of Obstetrics and Gynecology, Stanford University School of Medicine, Stanford, CA USA

**Keywords:** LGBTQ persons, Undergraduate medical education

## Abstract

**Purpose:**

To characterize current lesbian, gay, bisexual, transgender, queer, and intersex (LGBTQI +) health-related undergraduate medical education (UME) curricular content and associated changes since a 2011 study and to determine the frequency and extent of institutional instruction in 17 LGBTQI + health-related topics, strategies for increasing LGBTQI + health-related content, and faculty development opportunities.

**Method:**

Deans of medical education (or equivalent) at 214 allopathic or osteopathic medical schools in Canada and the United States were invited to complete a 36-question, Web-based questionnaire between June 2021 and September 2022. The main outcome measured was reported hours of LGBTQI + health-related curricular content.

**Results:**

Of 214 schools, 100 (46.7%) responded, of which 85 (85.0%) fully completed the questionnaire. Compared to 5 median hours dedicated to LGBTQI + health-related in a 2011 study, the 2022 median reported time was 11 h (interquartile range [IQR], 6–16 h, *p* < 0.0001). Two UME institutions (2.4%; 95% CI, 0.0%-5.8%) reported 0 h during the pre-clerkship phase; 21 institutions (24.7%; CI, 15.5%-33.9%) reported 0 h during the clerkship phase; and 1 institution (1.2%; CI, 0%-3.5%) reported 0 h across the curriculum. Median US allopathic clerkship hours were significantly different from US osteopathic clerkship hours (4 h [IQR, 1–6 h] *versus* 0 h [IQR, 0–0 h]; *p* = 0.01). Suggested strategies to increase content included more curricular material focusing on LGBTQI + health and health disparities at 55 schools (64.7%; CI, 54.6%-74.9%), more faculty willing and able to teach LGBTQI + -related content at 49 schools (57.7%; CI, 47.1%-68.2%), and more evidence-based research on LGBTQI + health and health disparities at 24 schools (28.2%; CI, 18.7%-37.8%).

**Conclusion:**

Compared to a 2011 study, the median reported time dedicated to LGBTQI + health-related topics in 2022 increased across US and Canadian UME institutions, but the breadth, efficacy, or quality of instruction continued to vary substantially. Despite the increased hours, this still falls short of the number of hours based on recommended LGBTQI + health competencies from the Association of American Medical Colleges. While most deans of medical education reported their institutions’ coverage of LGBTQI + health as ‘fair,’ ‘good,’ or ‘very good,’ there continues to be a call from UME leadership to increase curricular content. This requires dedicated training for faculty and students.

**Supplementary Information:**

The online version contains supplementary material available at 10.1186/s12909-024-05469-0.

## Background

Lesbian, gay, bisexual, transgender, queer, and intersex (LGBTQI +) health content during undergraduate medical education (UME) is an essential step toward reducing barriers to comprehensive and affirming healthcare for LGBTQI + populations [1]. With over 7% of the US adult population and 4% of the Canadian adult population identifying as LGBTQI + [2, 3], and over 20% of persons in the US 18–25 years-old identifying as LGBTQI + , physicians must be prepared to meet their health needs [4]. The geographical ubiquity of LGBTQI + persons and their experience of health disparities – including higher prevalence of tobacco, alcohol, substance use, and mental health concerns (e.g., anxiety, depression, suicidality) [1] as well as elevated cardiovascular disease morbidity and mortality [5, 6] – warrants explicitly including training in UME to prepare future clinicians [7].

LGBTQI + health-related content in UME can address the concerning rates of healthcare discrimination experienced by LGBTQI + persons. In the US, approximately 12% of lesbian, gay, and bisexuals individuals and over 20% of transgender and gender-diverse individuals reported being blamed for their health status when seeking healthcare [8]. Nearly 8% of lesbian, gay, and bisexual individuals and 29% of transgender and gender non-binary persons have been denied healthcare in the US [9]. A 2017 national survey of LGBTQI + US populations found that 16% of LGBTQI + people report being personally discriminated against because they are part of the LGBTQI + community when going to a doctor or health clinic; 22% of transgender individuals avoided doctors or healthcare out of concern they would be discriminated against; and 31% of transgender persons said they have no regular doctor or form of healthcare because of fear of discrimination [10]. In 2024, LGBTQI + adults were twice as likely as straight, cisgender adults to report negative experiences while receiving health care in the last three years, including being treated unfairly or with disrespect, having at least one of several other negative experiences with a provider, having a provider assume something about them without asking, suggesting they were personally to blame for a health problem, ignoring a direct request or question, or refusing to prescribe needed pain medication [11].

UME has included sparse instruction in LGBTQI + health-related content [12]. The 2011 study of US and Canadian allopathic and osteopathic medical schools by Obedin-Maliver et al*.*(data collected 2009–2010) reported a median 5 h of LGBTQI + health-related instruction with considerable variability in the training content and quality [13]. Since the 2011 study, there have been a number of initiatives to improve LGBTQI + UME that warrant a follow up study, including guidance from the Association of American Medical Colleges (AAMC), publication of curricular interventions on MedEdPortal, and dissemination of a AAMC video series on integrating LGBTQI + curricular content [14]. A 2017 study of 940 medical students at three US universities found, utilizing a validated measure of LGBTQI + competency [15], that students who cared for LGBTQI + patients or received ≥ 35 total hours of LGBTQI + health-related content reported significantly higher preparedness and knowledge to care for LGBTQI + patients [16]. These findings suggest that UME would benefit from increased time dedicated to LGBTQI + health instruction. Further, inclusion of validated measures of LGBTQI + competency in the assessment of graduates’ knowledge and preparedness to care for LGBTQI + persons would improve the evaluation and development of curricular interventions. A 2024 narrative review of LGBTQI + undergraduate medical education in the United States found multiple studies noting that students receive inadequate education, especially in their knowledge and preparedness to care for LGBTQI + patients, particularly transgender and gender diverse patients [12].

Despite recommendations, guidance, and explicit core competencies by the AAMC [14], questionnaires across specialties report little change in LGBTQI + health-related content in UME and continued knowledge deficits among recent graduates [17–20]. This study, a follow-up to the 2011 study by Obedin-Maliver et al*.* [13], aims to describe the current breadth and depth of LGBTQI + health-specific curricular content, the number of hours taught, coverage of relevant topics, strategies to increase content, faculty development opportunities, and deans' opinions of their institutions' LGBTQI + health-related content. Utilizing original data from the Obedin-Maliver et al*.* study, we describe changes in the median number of curricular hours dedicated to LGBTQI + health-related since 2011. Our primary hypothesis was that the median number of curricular hours dedicated to LGBTQI + health-related content had increased since 2011 and varied across institutions.

## Methods

### Study design

This study was a cross-sectional internet-based survey. The study was deemed exempt by the Boston University Medical Campus Institutional Review Board under 45 CFR 164.514 and electronic informed consent was obtained from participants. Prior data was accessed via a Data Use Agreement to perform comparative analytics on the 2011 data given by Stanford University, the site for the 2011 Obedin-Maliver et al*.* data collection and analytics.

### Questionnaire design and distribution

Questionnaire design relied on the prior survey conducted from 2009 to 2010 and published in 2011 with 150 of 176 schools (85.2%) responding, and 132 (75.0%) fully completing the questionnaire [13]. Questionnaire changes were made to reflect curricular and linguistic changes since original questionnaire distribution. Language regarding curricular phases was updated from “pre-clinical” and “clinical” (2011 study) to “pre-clerkship,” “clerkship,” and “post-clerkship” (present study) [21].

We maintained much of the 2011 questionnaire’s evaluation of LGBTQI + health-specific curricular content in pre-clerkship, clerkship, and post-clerkship periods and included an additional topic – intersectionality. The final 17 topics queried with “Do you cover the following health topics at your institution?” were not meant to be exhaustive but representative of critical LGBTQI + experiences that affect health and well-being. Additional questions evaluated how LGBTQI + health content is incorporated into the required and elective curricula, including assessing location of training (e.g., clerkship training options), and queried which resources deans of medical education (or their equivalent) believed are needed to improve training on LGBTQI + health. A questionnaire was drafted by the authorship team, which is composed of persons with LGBTQI + lived experience and expertise in clinical, research, and medical education regarding LGBTQI + matters. The authorship team utilized existing guidance (e.g., AAMC) as well as published literature on medical education initiatives. The drafted questionnaire was piloted with five deans of medical education (noted in Acknowledgements). Their feedback was incorporated into the final questionnaire. The final 36-item questionnaire was designed to be completed in 25–30 min (Supplemental Materials). Changes from the 2011 questionnaire are noted in eTable 1.

The final questionnaire was distributed between June 2021 and September 2022 to deans of medical education (or their equivalent) at all 172 allopathic medical schools (17 Canada, 155 United States) and all 42 osteopathic medical schools in the United States enrolling students at questionnaire initiation. The authors distributed initial invitations via e-mail. Repeated contacts with non-respondents were made nine times via email and once via telephone.

The questionnaire was administered via Qualtrics (Qualtrics Inc.; Seattle, WA, USA) with data encryption. Informed consent was obtained prior to starting the questionnaire. Only one questionnaire was requested from each institution. Unique links were generated for each UME institution, thereby avoiding the potential for multiple responses from an institution. All responses were collected without individual names, and school identities were kept confidential.

### Outcomes

The primary outcome was the number of UME curricular hours on LGBTQI + health-specific content. Secondary outcomes included the frequency and extent of institutional instruction in 17 LGBTQI + health-related topics, strategies for increasing LGBTQI + health-related content, and faculty development opportunities.

### Data coding and preparation for analysis

After the conclusion of data collection, responses were categorized by one research team member according to the American Association for Public Opinion Research (AAPOR) standards: complete with all questions answered (AAPOR code 1.1), incomplete with primary outcome questions answered (AAPOR code 1.2), or incomplete with primary outcome questions unanswered (AAPOR code 2.12) [22]. Completed (AAPOR code 1.1) and incomplete (AAPOR code 1.2) responses that answered the primary outcome questions were included in the primary analysis. Incomplete responses with primary outcome questions unanswered (AAPOR code 2.12) were utilized in analyses for which there were answers provided. For each additional analysis beyond the primary outcome, we utilized all available responses for each question. Non-responders were coded as AAPOR code 3.19. Responses from 2022 were confidentially matched by institution to 2011 responses for longitudinal analyses.

### Statistical analysis

Differences in pre-clerkship *versus* clerkship *versus* post-clerkship hours for all schools were compared using the Wilcoxon rank sum test. Institutions were categorized by country/degree type (Canadian allopathic, US allopathic, US osteopathic) and public/private institutional affiliation. Violations of assumptions necessary for parametric tests necessitated nonparametric testing (Kruskal–Wallis) to compare the reported pre-clerkship, clerkship, post-clerkship, and total hours. Due to unequal group sizes and large standard deviations between groups, medians were compared, but mean values for curricular hours were calculated for comparison with the 2011 study. Post hoc pairwise Mann–Whitney U tests with Bonferroni correction for multiple testing were performed on categories with significant (*p* < 0.05) Kruskal–Wallis results; Fisher exact test compared schools teaching a total of 0 *versus* more than 0 clerkship hours. These analyses are presented in Tables 1 and 2.

**Table 1:** Pre-clerkship, clerkship, post-clerkship, and total hours dedicated to LGBTQI + health-related topics in US and Canadian medical and osteopathic schools in 2022 (*N* = 85)

	**Pre-Clerkship Hours**		**Clerkship Hours**		**Post-Clerkship Hours**		**Total Hours**	
	**Median** **(IQR) [Range]**	***p*-value**	**Median** **(IQR) [Range]**	***p*-value**	**Median** **(IQR) [Range]**	***P*-value**	**Median** **(IQR) [Range]**	***P*-value**
**All (*n* = 85)**	8 (4–11) [0–42]		3 (1–6) [0–42]		0 (0–0) [0–8]		11 (6–16) [0–84]	
**Country**								
US (*n* = 80)	7 (4–10) [0–42]	0.046	3 (1–6) [0–42]	0.456	0 (0–0) [0–8]	0.0011	11 (6–16) [0–84]	0.308
Canada (*n* = 5)	12 (8–13 [8–30]		1 (1–4) [1–5]		3 (2–4) [0–6]		12 (12–14) [9–35]	
**Country and Degree**								
US allopathic (*n* = 70)	7 (4–10) [0–20]	0.101	4 (1–6) [0–12]	0.011	0 (0–0) [0–8]	0.002	11 (6–16) [2–28]	0.467
US osteopathic (*n* = 10)	9 (3–15) [0–42]		0 (0–0) [0–42]		0 (0–0) [0–0]		9 (3–15) [0–84]	
Canadian allopathic (*n* = 5)	12 (8–13) [8–30]		1 (1–4) [0–5]		3 (2–4) [0–6]		12 (12–14) [9–35]	
**Institution type**								
Private (*n* = 34)	7 (4–10) [0–42]	0.313	3 (0–4) [0–42]	0.583	0 (0–0) [0–8]	0.041	9 (5–14) [2–84]	0.164
Public (*n* = 51)	8 (4–12) [0–42]		3 (1–7) [0–12]		0 (0–2) [0–6]		12 (6–16) [0–46]

**Table 2:** Distribution of medical schools with zero versus more than zero hours dedicated to teaching LGBTQI + content 2022 (*n* = 92)

**Phase of Curriculum**	**0 Hours n (%)**	** > 0 Hours n (%)**	***P*-value**
Pre-clerkship	2 (2.3%)	86 (97.7%)	< .0001
Clerkship	21 (24.7%)	64 (75.3%)	
Post-Clerkship	61 (76.3%)	19 (23.8%)	
Combined	1 (1.1%)	91 (98.9%)	
**Pre-clerkship Hours Only by Category**			
**Country/Degree**	**0 Hours n (%)**	** > 0 Hours n (%)**	***P*-value**
Canadian Allopathic	0 (0%)	5 (5.7%)	0.3323
US Allopathic	1 (1.1%)	71 (80.7%)	
US Osteopathic	1 (1.1%)	10 (11.4%)	
**Affiliation**			
Private	1 (1.2%)	34 (41.0%)	1.0000
Public	1 (1.2%)	47 (56.7%)	
**Clerkship Hours Only by Category**			
**Country/Degree**	**0 Hours n (%)**	** > 0 Hours n (%)**	***P*-value**
Canadian Allopathic	1 (1.2%)	4 (4.7%)	< .0001
US Allopathic	12 (14.1%)	58 (68.2%)	
US Osteopathic	8 (9.4%)	2 (2.4%)	
**Affiliation**			
Private	9 (11.3%)	25 (31.3%)	0.7940
Public	11 (13.8%)	35 (43.8%)	
**Post-Clerkship Hours Only by Category**			
**Country/Degree**	**0 Hours n (%)**	** > 0 Hours n (%)**	***P*-value**
Canadian Allopathic	1 (1.3%)	4 (5.0%)	0.0063
US Allopathic	52 (65.0%)	15 (18.8%)	
US Osteopathic	8 (10.0%)	0 (0%)	
**Affiliation**			
Private	28 (37.3%)	4 (5.3%)	1.0000
Public	32 (42.7%)	11 (14.7%)	

^*^Percentages may not add to 100% because “don’t know” responses for either pre-clinical (*n* = 3) or clinical (*n* = 14) hours were removed from hours-related statistical analyses

Differences in the primary outcome (i.e., median hours) between institutions that completed questionnaires in 2011 and 2022 and those which completed only the 2011 questionnaire were evaluated utilizing Wilcoxon Rank Sum test. All secondary analyses conducted on continuous variables used the Wilcoxon signed-rank test where data were normally distributed; when assumptions of normality were violated, Kruskal–Wallis was used. All categorical or ordinal comparisons used Chi-squared tests of independence unless small cells were present, in which case Fishers exact tests were used. These analyses are presented in Table 3.

**Table 3:** Among institutions that completed the 2011 questionnaire (*n* = 132), there were no appreciable differences between responders (*n* = 60) and non-responders (*n* = 72) of the 2022 questionnaire

		**Responded to 2022 Questionnaire**			
		**NO, did not respond to 2022 questionnaire (*n* = 72)**	**YES, did respond to 2022 questionnaire (*n* = 60)**	***p*-value**	**Statistical Test**
**Hours reported in 2011**	**Median 2011 h (IQR) [Range]**	**Median 2011 h (IQR) [Range]**	**Median 2011 h (IQR) [Range]**		
Required Preclinical Hours	4 (2–6) [0–24]	4 (2–6) [0–20]	4 (2–6) [0–24]	0.8915	Wilcoxon Rank Sum
Required Clinical Hours	2 (0–3) [0–15]	2 (0–4) [0–15]	2 (0–3) [0–10]	0.7349	Wilcoxon Rank Sum
Total Required Hours	5 (3–8) [0–32]	5 (3–8) [0–32]	5 (3–8) [0–28]	0.8640	Wilcoxon Rank Sum
**Country**					
United States	121 (91.7%)	65 (90.3%)	56 (93.3%)	0.7535	Fisher's Exact
Canada	11 (8.3%)	7 (9.7%)	4 (6.7%)		
**Country and Degree**					
US Allopathic	102 (77.3%)	51 (70.8%)	51 (85.0%)	0.1337	Chi-Square
US Osteopathic	19 (14.4%)	14 (19.4%)	5 (8.3%)		
Canadian Allopathic	11 (8.3%)	7 (9.7%)	4 (6.7%)		
**Institution Type**					
Public	80 (60.6%)	43 (59.7%)	37 (61.7%)	0.8199	Chi-Square
Private	52 (39.4%)	29 (40.3%)	23 (38.3%)		

All statistical analyses were performed using SAS 9.4.

## Results

### Characterization of respondents and nonrespondents in 2022

Of 214 eligible UME institutions in 2022, 100 (46.7%) responded. Of these, 85 (85% of respondents) fully completed the questionnaire including primary outcome (AAPOR 1.1; complete responses); 15 (15% of respondents) did not report the primary outcome (AAPOR 2.1; incomplete responses). There were 114 non-responders (53.3% of all potential responses, AAPOR 3.19; no response received). Notably, participation was higher among US compared to Canadian UME institutions (47.7% *vs* 35.5%), US allopathic compared to US osteopathic UME institutions (52.9% *vs* 28.5%), and public compared to private UME institutions (65.1% *vs* 52.6%) (eTable 2).

### Hours of LGBTQI + health curricular content in 2022 and compared to 2011

In 2022, the median (11 h, interquartile range [IQR] 6–16 h) and mean (13 h) total hours were higher than in 2011 (*p* < 0.0001 for both comparisons). In 2022, pre-clerkship hours were significantly greater than the clerkship hours (8 h; IQR 4–11 h *vs* 3 h; IQR 1–6 h; *p* < 0.001). Additional details of hours by specific curricular phase are presented in Table 1.

In 2022, two UME institutions (2.4%; 95% CI 0%-5.6%) reported 0 h during pre-clerkship phases, 21 (24.7%; CI 15.5%-33.9%) reported 0 h during clerkship phases, and 61 (76.3%; CI 66.9%-85.1%) reported 0 h during post-clerkship phases with 1 (1.1%; CI 0%-3.2%) reporting 0 total hours. In 2022, Canadian allopathic (1; 1.2%; CI, 0.0%-55.1%) and US osteopathic (8; 9.4%; CI, 55.2%-100%) schools were more likely than US allopathic schools (12; 14.1%; CI, 8.3%-26.0%) to report 0 clerkship hours (*p* = 0.001) (Table 2). Compared to 2011, a smaller proportion of schools reported 0 total hours in 2022 (2.7% *vs* 0.45% *p* = 0.244).

### Differences in total hours of LGBTQI + health curricular content between responders and non-responders in 2022 compared to 2011

Among UME institutions that completed the 2011 questionnaire (*n* = 132), there were no significant differences of total hours of LGBTQI + health content reported by responders (*n* = 60) and non-responders (*n* = 72) to the 2022 questionnaire (Table 3). Further, the 2022 responders and non-responders who had completed the 2011 questionnaire did not differ significantly by country, degree conferred, institution type, or mean/median hours of LGBTQI + health curricular content (Table 3).

### Amount and quality of LGBTQI + health topic coverage in 2022 compared to 2011

In 2022, 80 UME institutions (94.1%; CI 89.1%-99.1%%) reported that they taught students the difference between sexual identity and sexual behavior; 2 (2.4%; CI 0%-5.58%) reported this was not taught, and 3 did not know whether this difference was taught (3.5%, CI 0%-7.5%). Compared to 2011, a greater proportion of UME institutions reported teaching students the difference between sexual identity and sexual behavior (71.2% *vs* 94.1% *p* < 0.0001).

**Fig. 1:**
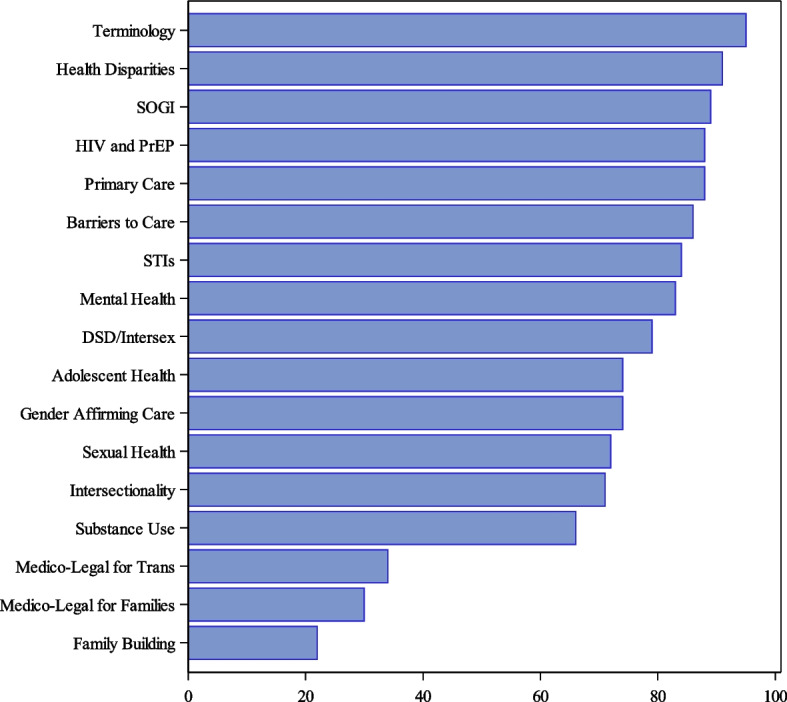
Proportion of 2022 respondents (*n* = 92) teaching LGBTQIA + Related Topics in the Required Curriculum. DSD: Differences in sex development; HIV: Human immunodeficiency virus; PrEP: Pre-exposure prophylaxis; SOGI: Sexual orientation and gender identity; STIs: sexually transmitted infections

In 2022, UME institutions reported the presence or absence of 17 LGBTQI + health-related topics in their required or elective curricula (Fig. 1).

**Fig. 2:**
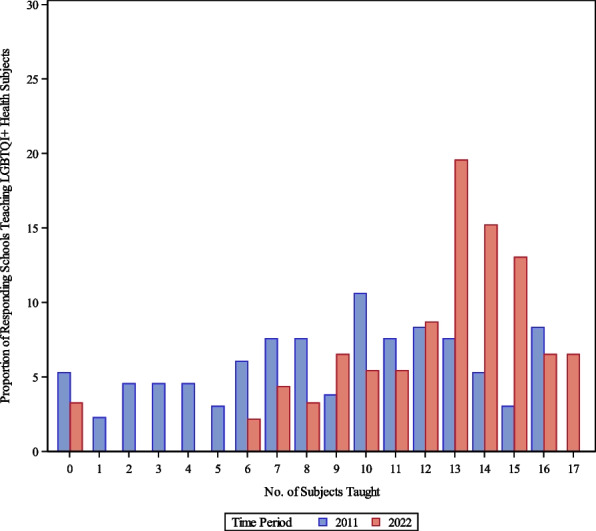
Cumulative Number of LGBTQI + Health-Related Topics Taught in the Required Curriculum, Comparing 2011 (*n* = 132) and 2022 (*n* = 92) Responses*. *2022 Survey had higher total number of topics possible (17 topics) than 2011 survey (16 topics)

The median number of LGBTQI + health-related topics taught in the required curriculum in 2022 was significantly higher, with 13 out of 17, compared to a median of 10 out of 16 in 2011 (*p* < 0.0001) (Fig. 2). In 2022, 6 (7.1%; CI 1.6%-12.5%) institutions reported teaching all 17 topics in their curricula (Fig. 2).

**Fig. 3:**
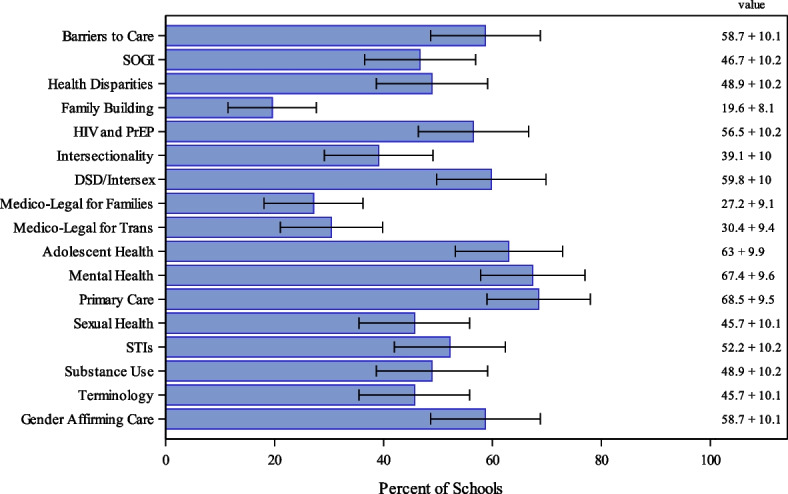
Proportion of 2022 respondents (*n* = 92) reporting “introductory (limited knowledge)” of each of 17 LGBTQI + -Related Topics at Their Institution. DSD: Differences in sex development; HIV: Human immunodeficiency virus; PrEP: Pre-exposure prophylaxis; SOGI: Sexual orientation and gender identity; STIs: sexually transmitted infections

In 2022, UME institutions reported a range of depth of coverage of the same 17 LGBTQI + health-related topics (Fig. 3). Notably, the percentage of deans reporting “introductory (limited knowledge)” of these topics ranged from 19.6% for “Family Building” to 68.5% for “Primary Care” for LGBTQI + persons (Fig. 3).

Deans reported their opinion of the quality of LGBTQI + health-related content overall in their UME institution. In 2022, the most common response was “Fair” in 38 UME institutions (44.7%; CI 34.1%-55.3%). A plurality of deans reported their institutions’ coverage of LGBTQI + health-related as “very good” or “good” (45.9%; CI 35.3%-36.5%). Compared to 2011, a smaller proportion of deans reported coverage as “very poor”, “poor” or “fair” in 2022 (69.7% vs 52.3%; *p* < 0.01).

### Faculty development on LGBTQI + health content in 2022 compared to 2011

In 2022, faculty development for teaching LGBTQI + health content was offered in 53 UME institutions (62.4%; CI 52.1%-72.7%); however, only 16 (18.8%; CI 10.5%-27.1%) reported it as mandatory for some or all faculty. Compared to 2011, more UME institutions reported offering any faculty development in 2022 (21.2% *vs* 62.4%, *p* < 0.0001).

### Methods of teaching LGBTQI + health topics in 2022 compared to 2011

In 2022, UME institutions reported LGBTQI + pre-clerkship content to be mostly “integrated (*i.e.,* thread) throughout the curriculum” (44; 51.8%; CI 41.4%-62.4%), followed by “taught in discrete periods (*i.e.,* modules, days, half-days) dedicated to LGBTQ content” (39; 45.9%; CI 35.3%-56.5%). A similar pattern was found in 2011, with LGBTQI + pre-clerkship content to be mostly "interspersed throughout various parts of the curriculum" (89; 67.4%; CI 59.4%-79.4%) followed by "taught in discrete modules dedicated to LGBT content" (31; 23.5%; CI 16.3%-30.7%). Compared to 2011, a larger proportion of UME institutions reported in 2022 teaching LGBTQI + pre-clerkship content across all methods (90.9% *vs* 97.6%, *p* = 0.0046).

In the clerkship and post-clerkship phases in 2022, most UME institutions (62; 72.9%; CI 63.5%-82.4%) used lectures or small groups to teach LGBTQI + health-related topics. Compared to 2011, a larger proportion of UME institutions reported in 2022 using lectures or small groups for teaching LGBTQI + topics in 2022 (59.1% *vs* 72.9%, *p* = 0.0004). In 2022, LGBTQI + health-focused clerkship sites were not offered in 28 UME institutions (32.9%; CI, 23.0%-42.9%); 22 (25.9%; CI, 16.6%—35.2%) only offered them as elective clerkship sites; 22 (25.9%; CI, 16.6%—35.2%) only offered them as required clerkship sites; and 6 schools offered them as both required and elective clerkship sites (7.1%; CI 1.6%—12.5%). Compared to 2011, a larger proportion of UME institutions offered any LGBTQI + clerkship sites (15.2% vs 58.8%, *p* < 0.0001).

### Methods for improving LGBTQI + health content in 2022

In 2022, the most popular reported strategies to increase LGBTQI + health-related curricular content were having access to: “curricular material focusing on LGBTQI + -related health and health disparities (62.0%),” “online, ready-made, physician-level course and associated curricular content on LGBTQI + health (58.7%),” and having “faculty willing and able to teach LGBTQI + -related curricular content (55.4%)” (Table 4).

**Table 4:** Strategies cited in 2022 that would help UME institutions to further ensure medical student learners have the knowledge, skills, and attitudes needed to provide competent LGBTQI + patient care (*N* = 92)

Strategy^a^	No. (%) [95% CI]
Curricular material focusing on LGBTQI + -related health/health disparities	57 (62.0) [52.0–71.9]
Online, ready-made, physician-level course and associated curricular content on LGBTQI + health	54 (58.7) [48.6–68.8]
Faculty willing and able to teach LGBTQI + -related curricular content	51 (55.4) [45.3–65.6]
More time in the curriculum to be able to teach LGBTQI + -related content	45 (48.9) [38.7–59.1]
Questions based on LGBTQI + health/health disparities on national examinations (e.g., USMLE)	44 (47.8) [37.6–58.0]
Learner assessments related to LGBTQI + -related knowledge, skills, and attitudes	42 (45.7) [35.5–55.8]
Access to LGBTQI + community members as standardized patients	37 (40.2) [30.2–50.2]
Increased financial resources	37 (40.2) [30.2–50.2]
Logistical support for teaching LGBTQI + -related curricular content	33 (35.9) [26.1–45.7]
Access to LGBTQI + -specific clinical sites	31 (33.7) [24.0–43.4]
Curricular material coverage required by accreditation bodies	30 (32.6) [23.0–42.2]
Access to LGBTQI + community members for patient panels	26 (28.3) [19.1–37.5]
More evidence-based research regarding LGBTQI + health/health disparities	25 (27.2) [18.1–36.3]
Something else (Free-text responses): • “Ensuring that the entire curriculum (not just "LGBTQ specific" ones) does not reinforce heteropatriarchy and cisnormativity.” • “We have good access noted in the last three areas, which is why I didn't check them, but we could always add more access to LGBTQI + specific clinical sites.” • “A standard or benchmark to guide content and assessment.” • “Resources to help with faculty development: Our faculty are generally willing but lack comfort/knowledge on the topics, and we do not have a lot of people who can do the trainings here.”	4 (4.3) [0.2–8.5]
Decline to answer	0 (0.0%) [0.0–0.0]

*CI* Confidence Interval, *LGBTQI +*  Lesbian, gay, bisexual, transgender, queer, intersex, *USMLE* United States Medical Licensing Exam

^a^Responses are from 2022 question “What strategies would help you to further ensure medical student learners have the knowledge, skills, and attitudes needed to provide competent LGBTQ patient care? (Please select all that apply)”

## Discussion

This study provides a contemporary estimate of the LGBTQI + health-related content in UME and its change since 2011 [13]. We found a significant increase in total curriculum hours as well as the number of LGBTQI + health-related topics covered by UME institutions. However, based on recommended competencies by the AAMC, the current reported median of 11 h falls short of the presumed number of curricular hours needed to provide excellent care to LGBTQI + patients [14, 16]. Further, compared to 2011, a larger proportion of deans reported coverage as “very good” or “good.” And despite this, deans of UME institutions continue to call for more curricular materials and competent faculty to teach LGBTQI + health-related topics.

Since highlighting the dearth of LGBTQI + health-related content in 2011 [13], despite recent anti-LGBTQI + legislation [23], there have been significant advances in LGBTQI + rights (*e.g.,* Obergefell v. Hodges; Bostock v. Clayton County, Georgia) and explicit inclusion of LGBTQI + persons in healthcare protections (*e.g.,*Affordable Care Act §1557) along with increased efforts to improve UME LGBTQI + health education. Additionally, some institutions have taken the additional step to develop concentrations or certificate programs in LGBTQI + health. A driver of improved UME LGBTQI + curricular content is an AAMC publication focused on LGBTQI + curricular changes [14]. Regulatory bodies and governmental agencies, including The Joint Commission [24] and US Department of Health and Human Services [25], also released comprehensive plans to improve LGBTQI + health by addressing training gaps which have helped spur changes.

Unfortunately, these gaps in UME curricula and outcomes are well-documented and persist in the face of calls to action [26]. Consequently, student comfort with caring for LGBTQI + populations has lagged [27, 28]. Similar gaps in knowledge and comfort have been reported internationally [29–31]. These reported gaps in knowledge and comfort are contemporary with our findings, suggesting that even a median of 11 h of LGBTQI + content is insufficient.

Curricular interventions to address these gaps typically take one of two forms. One form is encapsulated learning experiences, including dedicated didactic lectures, small group activities, or some combination of instructional methods used to create multi-session seminars [32–39]. The second form is an integrated approach with content and objectives being interwoven within existing curricular structures to enhance the visibility of LGBTQI + health topics and demonstrate the applicability of other health topics to LGBTQI + populations [40–43]. Our study notes the latter to be the more prevalent approach.

Our study found that LGBTQI + -related content was taught through a mix of required and elective curricular exposures. While adding LGBTQI + content to the required curriculum ensures uniform exposure, electives have a role in improving UME training [44–47]. Although electives are often attended by those with prior interest or exposure and may exclude those with the largest knowledge gaps, electives may be preliminary steps toward the eventual inclusion of content, objectives, instructional methods, or evaluations in required curricula. However, electives are frequently led by or developed with learners. While this affords learners with an opportunity to gain skills and potentially enrichen medical education experience, LGBTQI + health training is the responsibility of the institution and cannot be left to learners for standardization, sustainability, as well as quality of teaching and assessment; using unpaid labor frequently disproportionately burdens LGBTQI + learners.

Successful incorporation of LGBTQI + health into UME requires thoughtfully developed LGBTQI + health competencies and objectives with appropriate instructional methods and assessments to ensure mastery [48, 49]. While some schools have centrally coordinated this with curriculum reform efforts [47], most schools in this study chose to dedicate faculty effort to the governance and oversight of curricular “threads” [43]. There is no current best UME oversight/management practice to address LGBTQI + health; the optimal solution is likely institution- and context-dependent. Further, graduate medical education must continue and complement UME to ensure trainees receive specialty-specific training on LGBTQI + health [4].

Our study has several strengths. First, we had good participation and completion rates [22]. Second, respondents were well positioned to answer the questionnaire and well connected to institutional curricular teams to provide accurate details. Third, we attempted to minimize response and social desirability biases by assuring respondent and institutional confidentiality. Fourth, we administered this questionnaire over one academic year to minimize curricular variation. Finally, the longitudinal nature of this study allows for a more granular examination of LGBTQI + health-related curricular content change over time.

Our study has notable limitations. First, the full complement of participating UME institutions cannot be said to represent the entirety of UME institutions. Second, using reported instructional hours as a metric likely underestimated the total LGBTQI + health content due to the inability of a single quantitative measure to evaluate certain teaching modalities (*e.g.*, problem-based learning, standardized patients) as well as the subtle and integrated nature of clinical teaching. Third, because pre-clerkship curricular content is more frequently indexed and standardized than clerkship or post-clerkship content, the questionnaire may provide a more accurate accounting of pre-clerkship hours. Previous studies have used reported hours as an important quantitative measure across non-standardized curricula. Fourth, inaccurate recall and information biases may be present, reflecting the heterogeneity of instructors and materials over time. The number of instruction hours may not necessarily correlate with the breadth, efficacy, or quality of instruction. However, reported hours of instruction remain a uniform means of curricular comparison and are used by medical school accreditation bodies [21]. This study was designed to evaluate exposure to topics on a national level. Further work describing the effectiveness of such teaching in enhancing trainees' skills is essential.

While deans may have difficulty immediately recalling estimated LGBTQI + -specific curriculum hours, our questionnaire was designed to allow deans to designate who is best suited within their institution to complete the questionnaire. Additionally, evaluating the accuracy of deans’ (or their designees’) assessment of teaching and curricular content within their institution is of value in future research endeavors [7]. Similarly, statistical comparisons between the two time points may be complicated by the fact some of the responses are the same institution over time and may impart a degree of paired nature for the data. To evaluate the robustness of the Wilcoxon rank sum test to possible dependence from the partially paired sample, we ran a Wilcoxon signed-rank test (appropriate for paired samples) on the institutions that appear in both time points; the paired comparison reported a mean difference of 3.4 h (*p* < 0.0001). These paired results are similar to those we saw when comparing our main outcome. Further, given the typical changeover in personnel, deans, and curricula seen in over a decade, independence was assumed for all respondents.

## Conclusion

The median reported time dedicated to LGBTQI + health-related content in medical school in 2022 was 11 h, a significant increase of 6 h since 2011. The number of hours in the required curriculum, as well as number of LGBTQI + health-related topics covered, remains varied. While most deans of medical education reported their institutions’ coverage of LGBTQI + health as fair, good, or very good, deans reported the need for more strategies to increase curricular content including faculty training. Despite a statistically significant increase in the number of curricular hours regarding LGBTQI + health content from 5-h to 11-h, this falls short of the recommended number of curricular hours needed to provide excellent care to LGBTQI + patients [14, 16].

### Supplementary Information

**Supplementary Material 1.**

**Supplementary Material 2.**

## Data Availability

The datasets generated and/or analyzed during the current study are not publicly available due to participant consent process and agreement to not share without additional review. Datasets are available from the corresponding author on reasonable request following ethical review and data use agreement processes.

## Abbreviations

AAMC: Association of American Medical Colleges
AAPOR: American Association for Public Opinion Research
LGBTQI +: Lesbian, gay, bisexual, transgender, queer, and intersex
UME: Undergraduate medical education
