# Supplementary material for: Sexual and gender minority content in undergraduate medical education in the United States and Canada: current state and changes since 2011
Source: BMC Med Educ. 2024 May 1;24:482. doi: 10.1186/s12909-024-05469-0 (PMC11064371; doi:10.1186/s12909-024-05469-0)
Supplement: Supplementary file 1 — Supplementary Material 1. [file 12909_2024_5469_MOESM1_ESM.docx]

**eTable 1:** Side-by-side comparison of 2011 and 2022 questionnaires. Full 2022 questionnaire and response options are included in supplemental materials.

| **2011 Questionnaire** | **2022 Questionnaire** | **Modifications** |
| --- | --- | --- |
| Please choose your institution type. | Please choose your institution type. | Same between 2011 and 2022 |
|  | Please indicate the number of months in each REQUIRED curricular phase | New question in 2022 |
| How many TOTAL hours are dedicated to teaching LGBT content during the following phases of training? | How many TOTAL REQUIRED hours are dedicated to teaching LGBTQ content during the following phases of training? | Modified from “pre-clinical” and “clinical” in 2011 to “Pre-Clinical: Pre-Clerkship Phase”  “Clinical: Clerkship Phase”  “Clinical: Post-Clerkship Phase” in 2022 |
| In the REQUIRED PRE-CLINICAL curriculum, LGBT-specific content is ___________. | In the REQUIRED PRE-CLINICAL phase, LGBTQ-specific content is PRIMARILY… | Similar questions between 2011 and 2022 |
|  | Please describe how LGBTQ content is integrated in the pre-clerkship curriculum. | New question in 2022 |
|  | Please describe how LGBTQ content is taught in discrete periods in the pre-clerkship curriculum. | New question in 2022 |
|  | In the REQUIRED CLINICAL phase, LGBTQ-specific content is PRIMARILY… | New question in 2022 |
|  | Please describe how LGBTQ content is integrated in the clerkship curriculum. | New question in 2022 |
|  | Please describe how LGBTQ content is taught in discrete periods in the clerkship curriculum. | New question in 2022 |
| Does your institution have lectures or small-group sessions that include LGBT-specific content in the REQUIRED CLINICAL curriculum? | How is LGBTQ health incorporated into your institution’s REQUIRED curriculum? (Please select all that apply.) | 2011 question was modified in 2022 to allow for additional response options. |
|  | Please tell us more about how LGBTQ health is incorporated in your institution’s REQUIRED curriculum | New question in 2022 |
|  | How is LGBTQ health incorporated into your institution’s ELECTIVE curriculum? (Please check all that apply.) | New question in 2022 |
|  | Please tell us more about how LGBTQ health is incorporated in your institution’s ELECTIVE curriculum. | New question in 2022 |
| Is there a CLINICAL clerkship site that is specifically designed to facilitate LGBT patient care (*e.g.*, rotations in LGBT-focused care centers)? | Is there a CLINICAL site that includes an LGBTQ-focused patient care experience? | Similar questions between 2011 and 2022 |
|  | Please tell us more about the clinical site(s). | New question in 2022 |
|  | Which of the following components do you address in your REQUIRED curriculum regarding LGBTQ-specific health? (Please check all that apply.) | New question in 2022 |
| Does your institution provide faculty development for teaching about LGBT health? | Does your medical school provide **FACULTY DEVELOPMENT** to *educators* about LGBTQ health? (Please select all that apply.) | Similar question between 2011 and 2022 |
|  | Please provide details about who and how they receive LGBTQ health-specific faculty development | New question in 2022 |
|  | Does your institution (University, health system, etc.) **require** *faculty* (not just educators) development about LGBTQ health? | New question in 2022 |
| When learning how to conduct a sexual history, are students at your institution taught to obtain information about same-sex relations, *e.g.* asking “do you have sex with men, women, or both?” |  | Question removed in 2022 |
| Are medical students at your institution taught the difference between behavior and identity (*e.g.*, a man may have sex with other men and identify as straight)? | Are medical students at your institution taught the difference between sexual behavior and sexual identity (*e.g.*, a man may have sex with other men and identify as straight)? | Similar questions between 2011 and 2022 |
|  | Are medical students at your institution taught the difference between gender and sex? | New question in 2022 |
| Does your institution provide education for students in the following content areas at any point in the curriculum? | Do you cover the following health topics at your institution? | Similar question between 2011 and 2022. Intersectionality and intersex were added as an option in 2022. |
| Please describe your opinion of how the following content areas are covered at your institution | To what extent are the following content areas covered at your institution? | Similar question between 2011 and 2022. Intersectionality and intersex were added as an option in 2022. |
| Please describe your opinion on the coverage of LGBT content, on the whole, at your institution | Please describe your opinion on the coverage of LGBTQ content, on the whole, at your institution. | Similar question between 2011 and 2022. |
| Please list other LGBT-related topics that your institution provides or would like to provide. |  | Question removed in 2022 |
| What method(s) does your institution use to evaluate the efficacy of teaching LGBT-specific content to students? (Please check all that apply.) | Which methods does your institution use to ensure skill/competency attainment of LGBTQ-specific learning objectives by your learners? (Please select all that apply.) | Similar question between 2011 and 2022. |
|  | Please describe any LGBTQ-related curricular innovations or enhancements at your medical school (that you have not mentioned elsewhere in this survey). | New question in 2022 |
| What strategies do you think are or would be successful in increasing LGBT-specific content at your institution? (Please check all that apply.) | What strategies would help you to further ensure medical student learners have the knowledge, skills, and attitudes needed to provide competent LGBTQ patient care? (Please select all that apply.) | Similar question between 2011 and 2022 |
|  | Please describe how well you believe your curriculum PREPARES students to care for LGBTQ patients. | New question in 2022. |

**eTable 2**: Characterization of 2022 questionnaire response rates by nation/degree type and institutional affiliation

| **Survey Status (AAPOR Code)** | **Overall** | **Canada Allopathic** | **All U.S.** | **U.S. Allopathic** | **U.S. Osteopathic** | **Private** | **Public** |
| --- | --- | --- | --- | --- | --- | --- | --- |
| 1.1 (fully completed) | 85 (39.7%) | 5 (29.4%) | 80 (40.6%) | 71 (45.8%) | 9 (21.4%) | 34 (44.7%) | 46 (55.4%) |
| 2.1 (did not answer primary outcome) | 15 (7.0%) | 1 (5.9%) | 14 (7.1%) | 11 (7.1%) | 3 (7.1%) | 6 (7.9%) | 8 (9.7%) |
| 3.19 (non-responders) | 114 (53.3%) | 11 (64.7%) | 103 (52.3%) | 73 (47.1%) | 30 (71.4%) | 36 (47.4%) | 29 (34.9%) |
| Total | 214 (100%) | 17 (100%) | 197 (100%) | 155 (100%) | 42 (100%) | 76 (100%) | 83 (100%) |

AAPOR: American Association for Public Opinion Research

AAPOR Code 1.1: Fully answered questionnaire.

AAPOR Code 2.1: Incomplete questionnaires that did not answer the primary outcome question (i.e., reported number of pre-clerkship, clerkship, and post-clerkship hours).

AAPOR Code 3.19: Nothing ever returned.
