# Supplementary material for: Sexual and gender minority content in undergraduate medical education in the United States and Canada: current state and changes since 2011
Source: BMC Med Educ. 2024 May 1;24:482. doi: 10.1186/s12909-024-05469-0 (PMC11064371; doi:10.1186/s12909-024-05469-0)
Supplement: Supplementary file 2 — Supplementary Material 2. [file 12909_2024_5469_MOESM2_ESM.pdf]

Boston University

Default Question Block

We recommend completing this survey on a computer or tablet. Please do not use a smartphone as question formatting may display poorly on a small screen.

Welcome to the Lesbian, Gay, Bisexual, Transgender, and Queer Medical Education Assessment (LGBTQ-MEA)!

Thank you for your interest in this exciting research project designed to gather information that will help medical schools train healthcare professionals to provide excellent care to sexual and/or gender minority (SGM) people, which includes but is not limited to those who identify as lesbian, gay, bisexual, transgender, and queer (LGBTQ). In this study, we will use LGBTQ for convenience. This research project is an expansion of our work published nearly a decade ago in JAMA.

The current survey has been updated to reflect medical education developments and our own evolving sophistication concerning LGBTQ topics and issues. Each line was compared and contrasted with the previous survey to ensure comparability, with particular attention to survey questions associated with responses reported in previous survey findings. However, adaptations, subtractions, and related modifications were made to improve the accuracy and relevance of the current survey.

GLMA: Health Professionals Advancing LGBTQ Equality and the American Medical Association (AMA) have issued letters of support for the LGBTQ-MEA.

This survey is meant to be completed by a Dean of Medical Education (or equivalent).
If someone else is better suited to complete part or all of this survey, please forward the original email invitation you received to complete this survey. Please feel free to contact us at any time if you have questions.

This 36-question survey will ask you about the medical curriculum at your institution. We anticipate that this survey will take approximately 20-30 minutes. This survey focuses on how your institution trains medical students to care for lesbian, gay, bisexual, transgender, and queer (LGBTQ) individuals. We appreciate your time and energy in helping us understand the range of educational practices across medical schools.

Please be assured we will never share your email address or institution-specific answers. We will only be reporting data in aggregate. We will share any manuscripts produced from this research with all Association of American Medical Colleges (AAMC) and American Association of Colleges of Osteopathic Medicine (AACOM) member schools.

Please choose your institution type.

- Allopathic (MD-granting) school in Canada
- Allopathic (MD-granting) school in the United States
- Osteopathic (DO-granting) school in the United States

Please choose your institution.

If your institution is not listed, please select "Institution not listed" at the bottom of the list.

Please choose your institution.

If your institution is not listed, please select "Institution not listed" at the bottom of the list.

Please choose your institution.

If your institution is not listed, please select "Institution not listed" at the bottom of the list.

Please provide your institution name.

An informed consent detailing your rights as a research participant and our responsibilities to you as researchers is available here: LGBTQ MEA Approved Consent

Thank you again for contributing your time to this important and exciting endeavor.

- I certify that I have read the informed consent form. I understand my rights and responsibilities as well as those of the investigators as they have been presented here, and I affirm my wish to participate in this research study.
- I do not wish to participate in this research study.

Please indicate the number of months in each REQUIRED curricular phase.*

* Some institutions divide undergraduate medical education into “pre-clinical” and “clinical” phases. With the advent of early clinical experiences, the distinctions “pre-clerkship,” “clerkship,” and “post-clerkship” may be more appropriate. To accommodate different systems at different institutions, we list both here. The use of “Clinical Phase” in this survey refers to both “Clerkship” and “Post-Clerkship” phases.

Pre-Clinical: Pre-Clerkship Phase

Clinical: Clerkship Phase

Clinical: Post-Clerkship Phase

How many TOTAL REQUIRED hours are dedicated to teaching LGBTQ content during the following phases of training? (If your institution does not record content by the number of hours, please estimate as accurately as possible.)

Pre-Clinical: Pre-Clerkship Phase

Clinical: Clerkship Phase

Clinical: Post-Clerkship Phase

Please complete the following statement:
In the REQUIRED PRE-CLINICAL phase, LGBTQ-specific content is PRIMARILY…

- Integrated (i.e., thread) throughout the curriculum (please describe below)

- ◯ Taught in discrete periods (i.e., modules, days, half-days) dedicated to LGBTQ content (please describe below)
- ◯ Not taught
- ◯ Don't know
- ◯ Decline to answer

Please describe how LGBTQ content is integrated in the pre-clerkship curriculum.

Please describe how LGBTQ content is taught in discrete periods in the pre-clerkship curriculum.

Please complete the following statement:
In the REQUIRED CLINICAL phase, LGBTQ-specific content is PRIMARILY…

- ◯ Integrated (i.e., thread) throughout the curriculum (please describe below)
- ◯ Taught in discrete periods (i.e., modules, days, half-days) dedicated to LGBTQ content (please describe below)
- ◯ Not taught
- ◯ Don't know
- ◯ Decline to answer

Please describe how LGBTQ content is integrated in the clerkship curriculum.

Please describe how LGBTQ content is taught in discrete periods in the clerkship curriculum.

Is there a CLINICAL site that includes an LGBTQ-focused patient care experience? (Please select all that apply.)

- ☐ Yes, this is a required experience for all students
- ☐ Yes, this is a site option as part of a required clerkship
- ☐ Yes, this is an elective clerkship
- ☐ No
- ☐ Don’t know
- ☐ Decline to answer

Please tell us more about the clinical site(s).

How is LGBTQ health incorporated into your institution’s REQUIRED curriculum? (Please select all that apply.)

	Pre-Clerkship Phase	Clerkship/Post-Clerkship Phase
Lecture(s)	☐	☐

	Pre-Clerkship Phase	Clerkship/Post-Clerkship Phase
Small Group(s)	☐	☐
Dedicated Module(s)	☐	☐
Standardized Patient(s)	☐	☐
Online Module(s)	☐	☐
Other (please describe below)	☐	☐
Not taught	☐	☐
Decline to answer	☐	☐

Please tell us more about how LGBTQ health is incorporated in your institution's REQUIRED curriculum.

How is LGBTQ health incorporated into your institution's ELECTIVE curriculum? (Please check all that apply.)

	Pre-Clerkship Phase	Clerkship/Post-Clerkship Phase
Lecture(s)	☐	☐
Small Group(s)	☐	☐
Dedicated Module(s)	☐	☐
Standardized Patient(s)	☐	☐
Online Module(s)	☐	☐
Clinical Experience/Rotation	☐	☐
Other (please describe below)	☐	☐
Not taught	☐	☐
Decline to answer	☐	☐

Please tell us more about how LGBTQ health is incorporated in your institution's ELECTIVE curriculum.

Which of the following components do you address in your REQUIRED curriculum regarding LGBTQ-specific health? (Please check all that apply.)

- ☐ Knowledge - (e.g., Medical Knowledge, Knowledge for Practice)
- ☐ Clinical Skills - (e.g., Patient Care, Communications, Systems-Based Practice)
- ☐ Attitudes - (e.g., Professionalism, etc.)
- ☐ Don’t know
- ☐ Decline to answer

Are medical students at your institution taught the difference between sexual behavior and sexual identity (*e.g.*, a man may have sex with other men and identify as straight)?

- ○ Yes
- ○ No
- ○ Don’t know
- ○ Decline to answer

Are medical students at your institution taught the difference between gender and sex?

Gender: the social and cultural constructs related to specific roles and behavior of men, women, and people of other genders.
Sex: physiologic, anatomic, genetic, and chromosomal components of males, females, and people with differences of sex development (DSD)/intersex.

- ○ Yes
- ○ No
- ○ Don’t know
- ○ Decline to answer

Please describe your opinion on the coverage of LGBTQ content, on the whole, at your institution.

- ○ Very Good
- ○ Good
- ○ Fair
- ○ Poor
- ○ Very Poor
- ○ Don’t know
- ○ Decline to answer

Which methods does your institution use to ensure skill/competency attainment of LGBTQ-specific learning objectives by your learners? (Please select all that apply.)

- ☐ Written examination(s)/assignment(s)
- ☐ Faculty-observed patient interactions
- ☐ Faculty-observed simulation interactions [observed structured clinical examination (OSCE), standardized patient]
- ☐ Student peer-to-peer evaluation (simulation, role play, patient encounter)
- ☐ Self-evaluations
- ☐ Evaluation by patients
- ☐ ________ Other
- ☐ Does not evaluate
- ☐ Decline to answer

Please describe how well you believe your curriculum PREPARES students to care for LGBTQ patients.

- ○ Very well
- ○ Well
- ○ Adequately
- ○ Poorly
- ○ Very Poorly
- ○ Don’t know
- ○ Decline to answer

Do you cover the following health topics at your institution?

	Yes	No	Don't know	Decline to answer
Primary care of LGBTQ patients	○	○	○	○
LGBTQ adolescent health	○	○	○	○
Mental health in LGBTQ individuals	○	○	○	○
Terminology regarding LGBTQ individuals	○	○	○	○
Documenting sexual orientation and gender identity in the medical record	○	○	○	○
Alcohol, tobacco, or other drug use among LGBTQ individuals	○	○	○	○
Health disparities in LGBTQ Communities	○	○	○	○
Family building in LGBTQ patients	○	○	○	○
Sexuality & sexual health (excluding sexually-transmitted infections, STIs) for LGBTQ people (including sexual satisfaction)	○	○	○	○
HIV and HIV pre-exposure prophylaxis (PrEP) among LGBTQ people	○	○	○	○
Sexually-transmitted infections (excluding HIV) among LGBTQ people	○	○	○	○
Gender-affirming health care for transgender patients (*i.e.*, hormones, surgery, etc.)	○	○	○	○
Differences in sex development (DSD)/Intersex	○	○	○	○
Medico-legal considerations for LGBTQ families (*i.e.*, spousal rights, parental rights, etc.)	○	○	○	○
Medico-legal considerations for transgender patients (*i.e.*, change government ID gender markers, insurance non-discrimination policies, etc.)	○	○	○	○
Barriers to accessing medical care for LGBTQ individuals	○	○	○	○
Intersectionality * <br>* Intersectionality is the complex, cumulative way in which the effects of multiple forms of discrimination (such as racism, sexism, homophobia, transphobia) combine, overlap, or intersect, especially in the experiences of marginalized individuals or groups (e.g., transgender women of color)	○	○	○	○

To what extent are the following content areas covered at your institution?

	Introductory (Limited Knowledge)	Advanced (In-Depth Knowledge)	Don’t Know to What Extent Content is Covered
Primary care of LGBTQ patients	○	○	○
LGBTQ adolescent health	○	○	○
Mental health in LGBTQ individuals	○	○	○
Terminology regarding LGBTQ individuals	○	○	○
Documenting sexual orientation and gender identity in the medical record	○	○	○
Alcohol, tobacco, or other drug use among LGBTQ individuals	○	○	○
Health disparities in LGBTQ Communities	○	○	○
Family building in LGBTQ patients	○	○	○
Sexuality & sexual health (excluding sexually-transmitted infections, STIs) for LGBTQ people (including sexual satisfaction)	○	○	○
HIV and HIV pre-exposure prophylaxis (PrEP) among LGBTQ people	○	○	○
Sexually-transmitted infections (excluding HIV) among LGBTQ people	○	○	○
Gender-affirming health care for transgender patients (*i.e.*, hormones, surgery, etc.)	○	○	○
Differences in sex development (DSD)/Intersex	○	○	○
Medico-legal considerations for LGBTQ families (*i.e.*, spousal rights, parental rights, etc.)	○	○	○
Medico-legal considerations for transgender patients (*i.e.*, change government ID gender markers, insurance non-discrimination policies, etc.)	○	○	○
Barriers to accessing medical care for LGBTQ individuals	○	○	○

	Introductory (Limited Knowledge)	Advanced (In-Depth Knowledge)	Don't Know to What Extent Content is Covered
Intersectionality * <br>* Intersectionality is the complex, cumulative way in which the effects of multiple forms of discrimination (such as racism, sexism, homophobia, transphobia) combine, overlap, or intersect, especially in the experiences of marginalized individuals or groups (e.g., transgender women of color)	○	○	○

Does your medical school provide **FACULTY DEVELOPMENT** to *educators* about LGBTQ health? (Please select all that apply.)

- ☐ Yes, mandatory for all educators (Please describe below.)
- ☐ Yes, mandatory for select educators (Please describe below.)
- ☐ Yes, optional (Please describe below.)
- ☐ No
- ☐ Don't know
- ☐ Decline to answer.

Please provide details about who and how they receive LGBTQ health-specific faculty development.

Does your institution (University, health system, etc.) **require** *faculty* (not just educators) development about LGBTQ health?

- ○ Yes
- ○ No
- ○ Don't know
- ○ Decline to answer

What strategies would help you to further ensure medical student learners have the knowledge, skills, and attitudes needed to provide competent LGBTQ patient care? (Please select all that apply.)

- ☐ Curricular material focusing on LGBTQ-related health/health disparities
- ☐ Online, ready-made, physician-level course and associated curricular content on LGBTQ health
- ☐ Faculty willing and able to teach LGBTQ-related curricular content
- ☐ Increased financial resources
- ☐ Staff support for teaching LGBTQ-related curricular content
- ☐ More time in the curriculum to be able to teach LGBTQ-related content
- ☐ More evidence-based research regarding LGBTQ health/health disparities
- ☐ Curricular material coverage required by accreditation bodies
- ☐ Questions based on LGBTQ health/health disparities on national examinations (*e.g.*, USMLE)
- ☐ Learner assessments related to LGBTQ-related knowledge, skills, and attitudes
- ☐ Access to LGBTQ community members as standardized patients
- ☐ Access to LGBTQ community members for patient panels
- ☐ Access to LGBTQ-specific clinical sites
- ☐ ______ Other (please specify)
- ☐ Decline to answer

Please describe any LGBTQ-related curricular innovations or enhancements at your medical school (that you have not mentioned elsewhere in this survey).

______

Please provide the following information.

Reminder: We use your contact information only to follow-up with you. We will not report data about individual institutions.

First Name ______

Last Name

Institutional Title

E-mail Address

Telephone Number
(###-###-####)

Powered by Qualtrics
